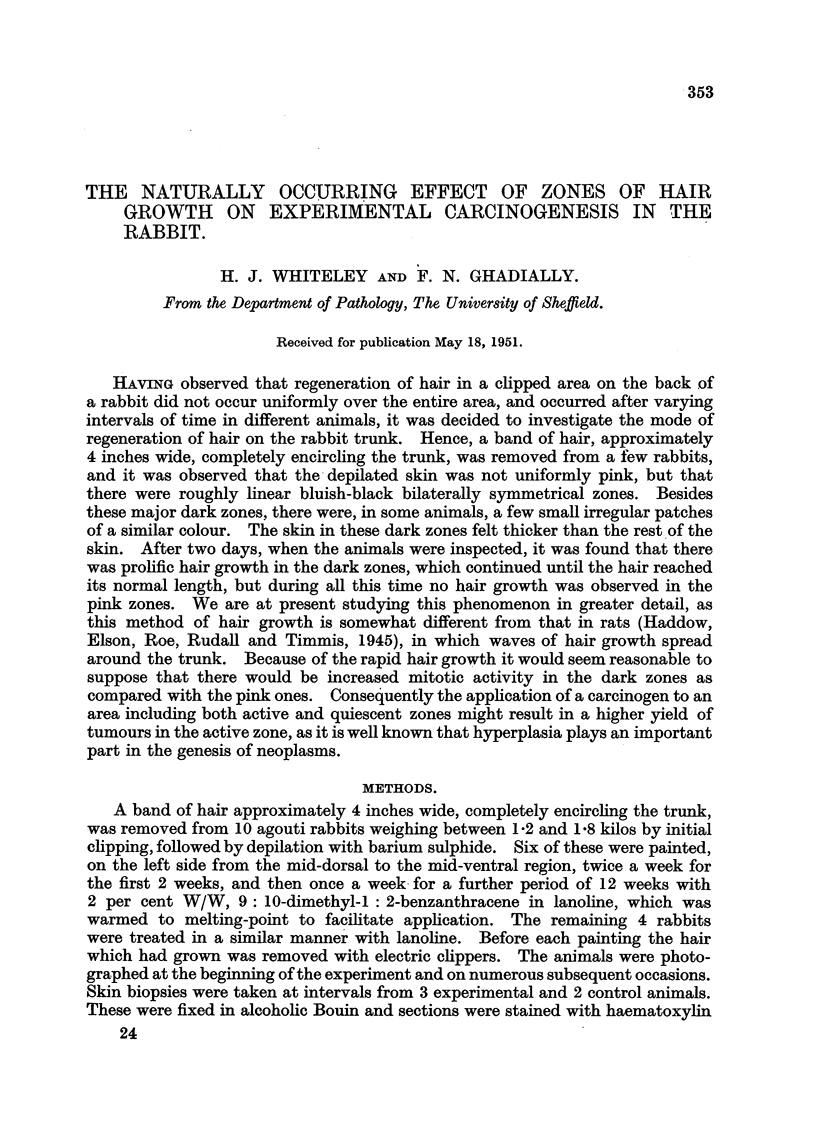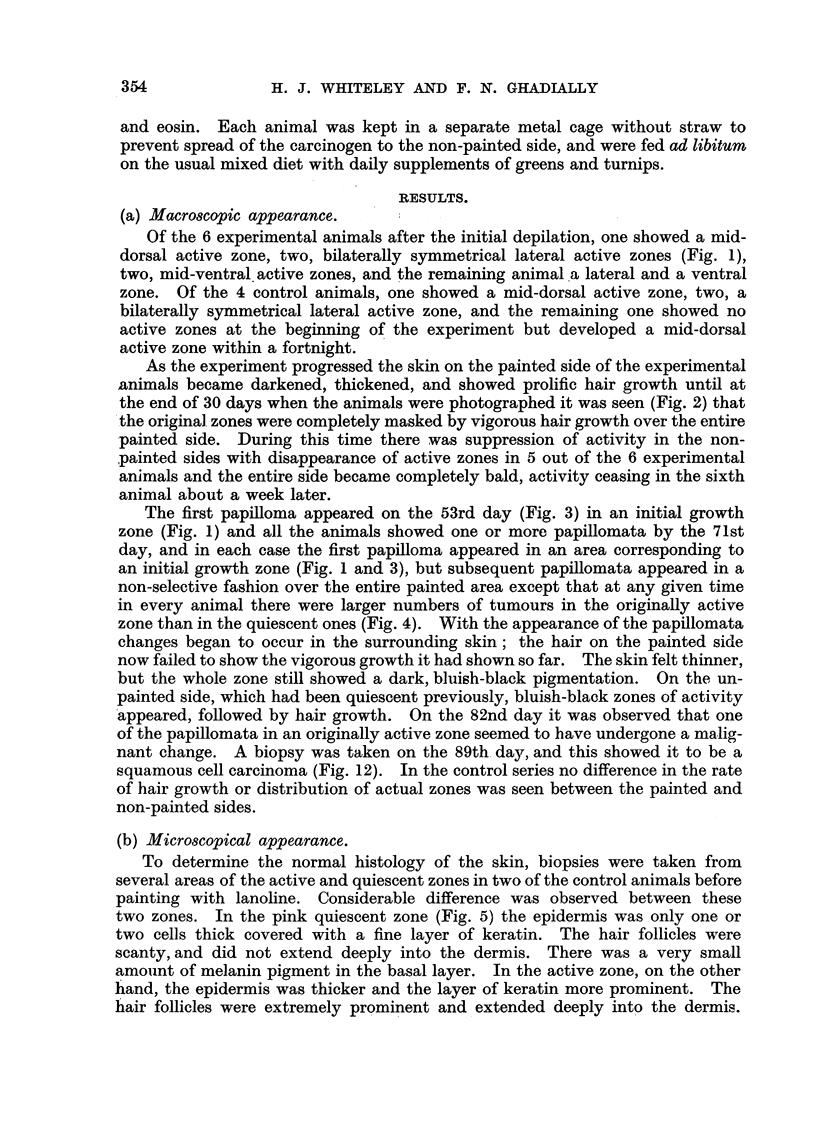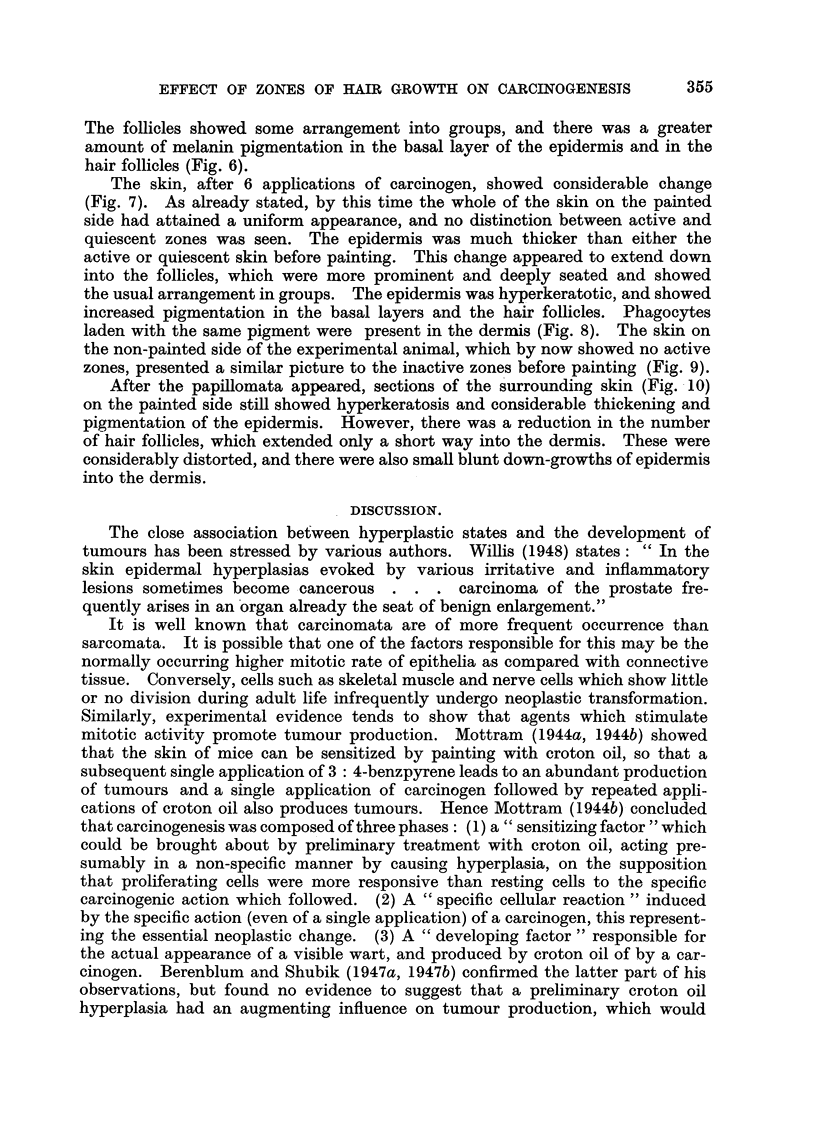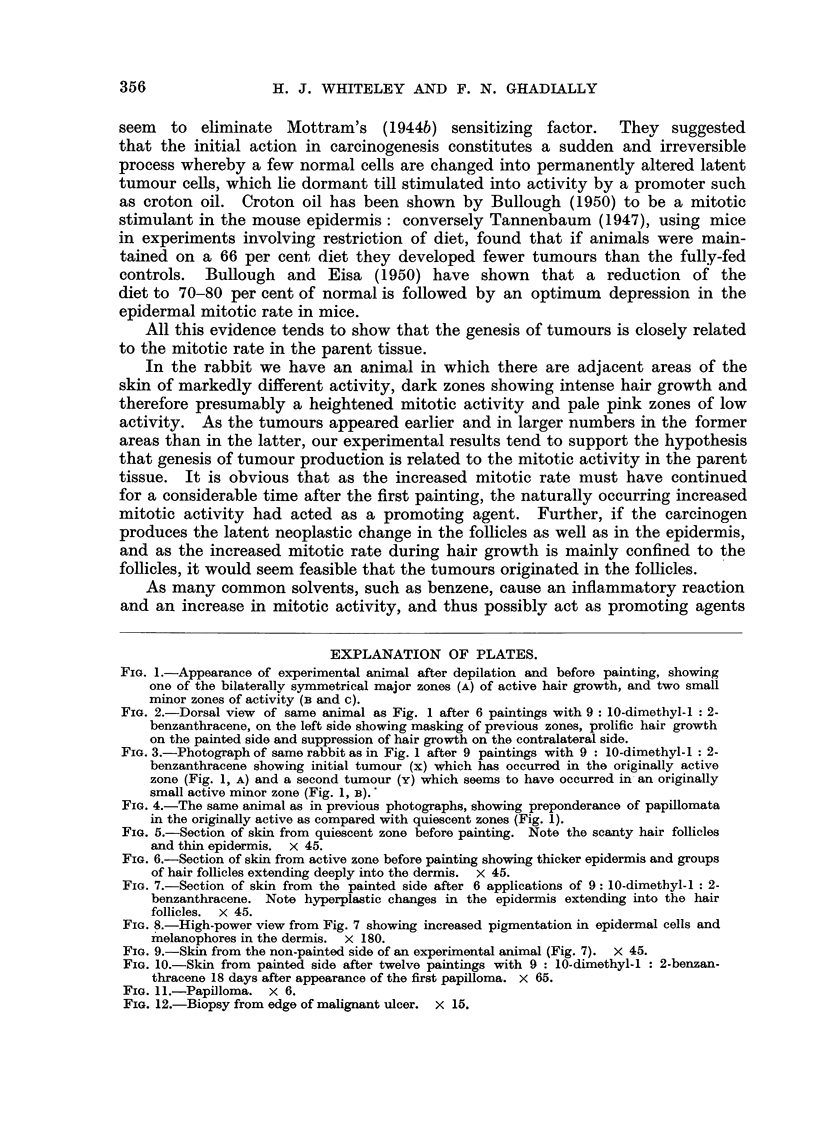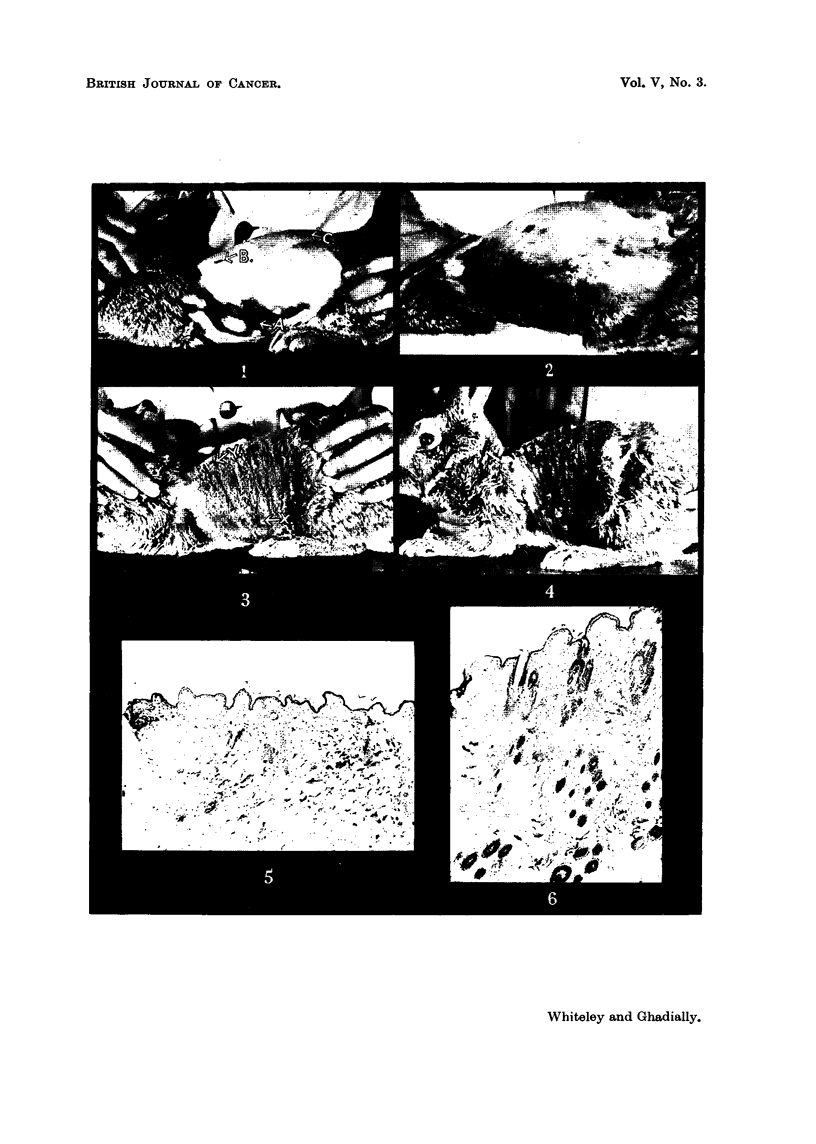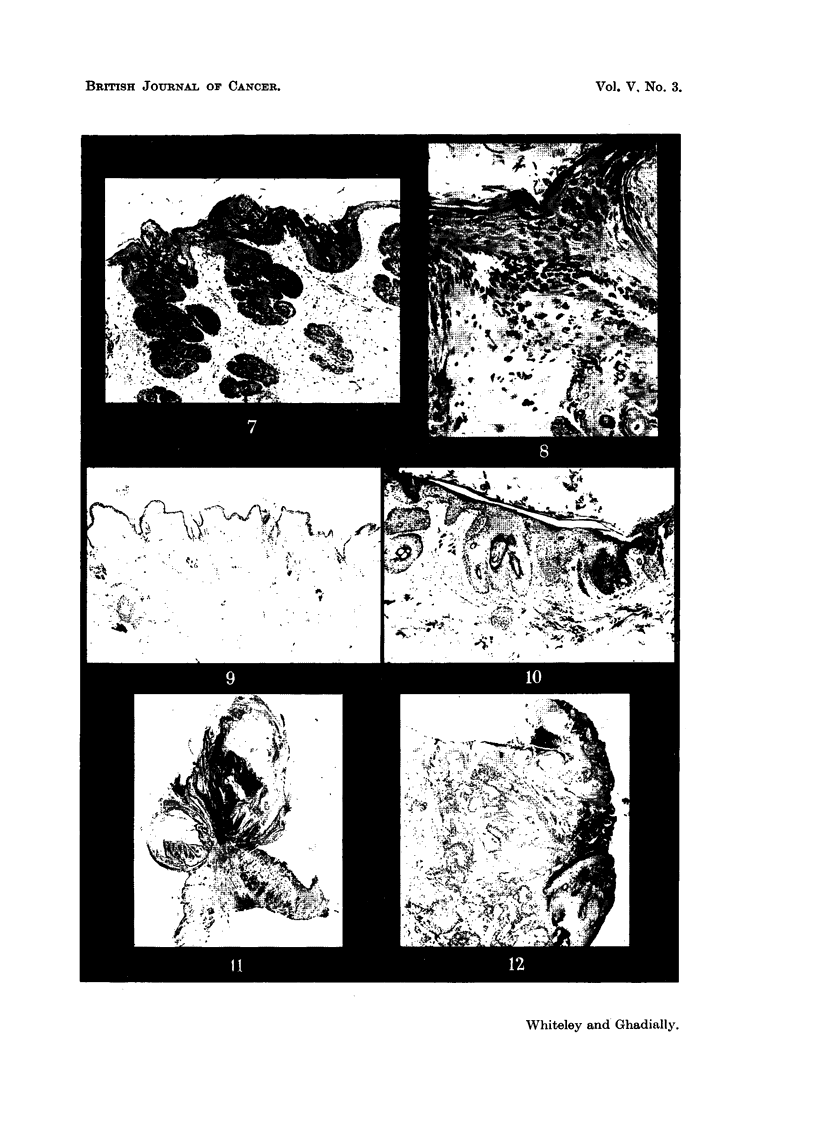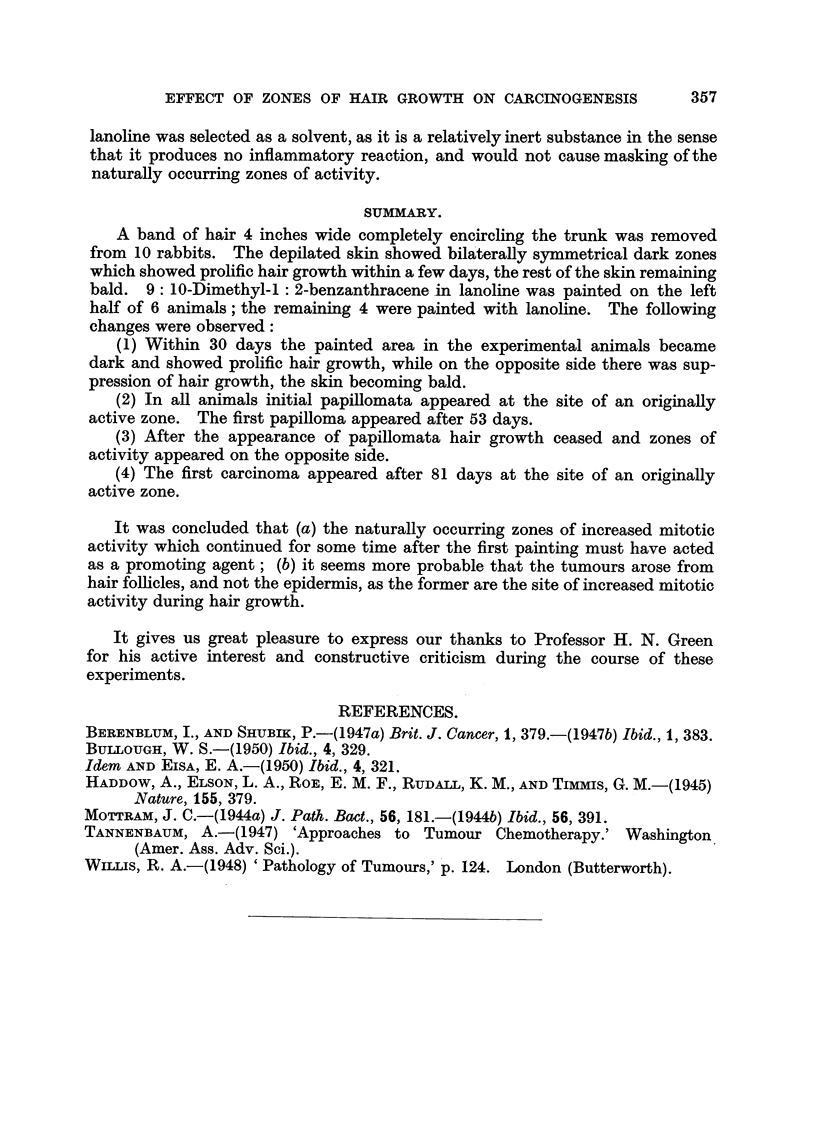# The Naturally Occurring Effect of Zones of Hair Growth on Experimental Carcinogenesis in the Rabbit

**DOI:** 10.1038/bjc.1951.37

**Published:** 1951-09

**Authors:** H. J. Whiteley, F. N. Ghadially

## Abstract

**Images:**


### THE NATURALLY OCCURRING EFFECT OF ZONES OF HAIR GROWTH ON EXPERIMENTAL CARCINOGENESIS IN THE RABBIT.

H. J. WHITELEY AND F. N. GHADIALLY.

*From the Department of Pathology, The University of Sheffield.*

Received for publication May 18, 1951.

HAVING observed that regeneration of hair in a clipped area on the back of a rabbit did not occur uniformly over the entire area, and occurred after varying intervals of time in different animals, it was decided to investigate the mode of regeneration of hair on the rabbit trunk. Hence, a band of hair, approximately 4 inches wide, completely encircling the trunk, was removed from a few rabbits, and it was observed that the depilated skin was not uniformly pink, but that there were roughly linear bluish-black bilaterally symmetrical zones. Besides these major dark zones, there were, in some animals, a few small irregular patches of a similar colour. The skin in these dark zones felt thicker than the rest of the skin. After two days, when the animals were inspected, it was found that there was prolific hair growth in the dark zones, which continued until the hair reached its normal length, but during all this time no hair growth was observed in the pink zones. We are at present studying this phenomenon in greater detail, as this method of hair growth is somewhat different from that in rats (Haddow, Elson, Roe, Rudall and Timmis, 1945), in which waves of hair growth spread around the trunk. Because of the rapid hair growth it would seem reasonable to suppose that there would be increased mitotic activity in the dark zones as compared with the pink ones. Consequently the application of a carcinogen to an area including both active and quiescent zones might result in a higher yield of tumours in the active zone, as it is well known that hyperplasia plays an important part in the genesis of neoplasms.

### METHODS.

A band of hair approximately 4 inches wide, completely encircling the trunk, was removed from 10 agouti rabbits weighing between 1·2 and 1·8 kilos by initial clipping, followed by depilation with barium sulphide. Six of these were painted, on the left side from the mid-dorsal to the mid-ventral region, twice a week for the first 2 weeks, and then once a week for a further period of 12 weeks with 2 per cent W/W, 9 : 10-dimethyl-1 : 2-benzanthracene in lanoline, which was warmed to melting-point to facilitate application. The remaining 4 rabbits were treated in a similar manner with lanoline. Before each painting the hair which had grown was removed with electric clippers. The animals were photographed at the beginning of the experiment and on numerous subsequent occasions. Skin biopsies were taken at intervals from 3 experimental and 2 control animals. These were fixed in alcoholic Bouin and sections were stained with haematoxylin and eosin. Each animal was kept in a separate metal cage without straw to prevent spread of the carcinogen to the non-painted side, and were fed *ad libitum* on the usual mixed diet with daily supplements of greens and turnips.

### RESULTS.

(a) *Macroscopic appearance.*

Of the 6 experimental animals after the initial depilation, one showed a mid-dorsal active zone, two, bilaterally symmetrical lateral active zones (Fig. 1), two, mid-ventral active zones, and the remaining animal a lateral and a ventral zone. Of the 4 control animals, one showed a mid-dorsal active zone, two, a bilaterally symmetrical lateral active zone, and the remaining one showed no active zones at the beginning of the experiment but developed a mid-dorsal active zone within a fortnight.

As the experiment progressed the skin on the painted side of the experimental animals became darkened, thickened, and showed prolific hair growth until at the end of 30 days when the animals were photographed it was seen (Fig. 2) that the original zones were completely masked by vigorous hair growth over the entire painted side. During this time there was suppression of activity in the non-painted sides with disappearance of active zones in 5 out of the 6 experimental animals and the entire side became completely bald, activity ceasing in the sixth animal about a week later.

The first papilloma appeared on the 53rd day (Fig. 3) in an initial growth zone (Fig. 1) and all the animals showed one or more papillomata by the 71st day, and in each case the first papilloma appeared in an area corresponding to an initial growth zone (Fig. 1 and 3), but subsequent papillomata appeared in a non-selective fashion over the entire painted area except that at any given time in every animal there were larger numbers of tumours in the originally active zone than in the quiescent ones (Fig. 4). With the appearance of the papillomata changes began to occur in the surrounding skin ; the hair on the painted side now failed to show the vigorous growth it had shown so far. The skin felt thinner, but the whole zone still showed a dark, bluish-black pigmentation. On the unpainted side, which had been quiescent previously, bluish-black zones of activity appeared, followed by hair growth. On the 82nd day it was observed that one of the papillomata in an originally active zone seemed to have undergone a malignant change. A biopsy was taken on the 89th day, and this showed it to be a squamous cell carcinoma (Fig. 12). In the control series no difference in the rate of hair growth or distribution of actual zones was seen between the painted and non-painted sides.

(b) *Microscopical appearance.*

To determine the normal histology of the skin, biopsies were taken from several areas of the active and quiescent zones in two of the control animals before painting with lanoline. Considerable difference was observed between these two zones. In the pink quiescent zone (Fig. 5) the epidermis was only one or two cells thick covered with a fine layer of keratin. The hair follicles were scanty, and did not extend deeply into the dermis. There was a very small amount of melanin pigment in the basal layer. In the active zone, on the other hand, the epidermis was thicker and the layer of keratin more prominent. The hair follicles were extremely prominent and extended deeply into the dermis.

The follicles showed some arrangement into groups, and there was a greater amount of melanin pigmentation in the basal layer of the epidermis and in the hair follicles (Fig. 6).

The skin, after 6 applications of carcinogen, showed considerable change (Fig. 7). As already stated, by this time the whole of the skin on the painted side had attained a uniform appearance, and no distinction between active and quiescent zones was seen. The epidermis was much thicker than either the active or quiescent skin before painting. This change appeared to extend down into the follicles, which were more prominent and deeply seated and showed the usual arrangement in groups. The epidermis was hyperkeratotic, and showed increased pigmentation in the basal layers and the hair follicles. Phagocytes laden with the same pigment were present in the dermis (Fig. 8). The skin on the non-painted side of the experimental animal, which by now showed no active zones, presented a similar picture to the inactive zones before painting (Fig. 9).

After the papillomata appeared, sections of the surrounding skin (Fig. 10) on the painted side still showed hyperkeratosis and considerable thickening and pigmentation of the epidermis. However, there was a reduction in the number of hair follicles, which extended only a short way into the dermis. These were considerably distorted, and there were also small blunt down-growths of epidermis into the dermis.

### DISCUSSION.

The close association between hyperplastic states and the development of tumours has been stressed by various authors. Willis (1948) states : " In the skin epidermal hyperplasias evoked by various irritative and inflammatory lesions sometimes become cancerous . . . carcinoma of the prostate frequently arises in an organ already the seat of benign enlargement."

It is well known that carcinomata are of more frequent occurrence than sarcomata. It is possible that one of the factors responsible for this may be the normally occurring higher mitotic rate of epithelia as compared with connective tissue. Conversely, cells such as skeletal muscle and nerve cells which show little or no division during adult life infrequently undergo neoplastic transformation. Similarly, experimental evidence tends to show that agents which stimulate mitotic activity promote tumour production. Mottram (1944*a*, 1944*b*) showed that the skin of mice can be sensitized by painting with croton oil, so that a subsequent single application of 3 : 4-benzpyrene leads to an abundant production of tumours and a single application of carcinogen followed by repeated applications of croton oil also produces tumours. Hence Mottram (1944*b*) concluded that carcinogenesis was composed of three phases : (1) a " sensitizing factor " which could be brought about by preliminary treatment with croton oil, acting presumably in a non-specific manner by causing hyperplasia, on the supposition that proliferating cells were more responsive than resting cells to the specific carcinogenic action which followed. (2) A " specific cellular reaction " induced by the specific action (even of a single application) of a carcinogen, this representing the essential neoplastic change. (3) A " developing factor " responsible for the actual appearance of a visible wart, and produced by croton oil of by a carcinogen. Berenblum and Shubik (1947*a*, 1947*b*) confirmed the latter part of his observations, but found no evidence to suggest that a preliminary croton oil hyperplasia had an augmenting influence on tumour production, which would seem to eliminate Mottram's (1944*b*) sensitizing factor. They suggested that the initial action in carcinogenesis constitutes a sudden and irreversible process whereby a few normal cells are changed into permanently altered latent tumour cells, which lie dormant till stimulated into activity by a promoter such as croton oil. Croton oil has been shown by Bullough (1950) to be a mitotic stimulant in the mouse epidermis : conversely Tannenbaum (1947), using mice in experiments involving restriction of diet, found that if animals were maintained on a 66 per cent diet they developed fewer tumours than the fully-fed controls. Bullough and Eisa (1950) have shown that a reduction of the diet to 70–80 per cent of normal is followed by an optimum depression in the epidermal mitotic rate in mice.

All this evidence tends to show that the genesis of tumours is closely related to the mitotic rate in the parent tissue.

In the rabbit we have an animal in which there are adjacent areas of the skin of markedly different activity, dark zones showing intense hair growth and therefore presumably a heightened mitotic activity and pale pink zones of low activity. As the tumours appeared earlier and in larger numbers in the former areas than in the latter, our experimental results tend to support the hypothesis that genesis of tumour production is related to the mitotic activity in the parent tissue. It is obvious that as the increased mitotic rate must have continued for a considerable time after the first painting, the naturally occurring increased mitotic activity had acted as a promoting agent. Further, if the carcinogen produces the latent neoplastic change in the follicles as well as in the epidermis, and as the increased mitotic rate during hair growth is mainly confined to the follicles, it would seem feasible that the tumours originated in the follicles.

As many common solvents, such as benzene, cause an inflammatory reaction and an increase in mitotic activity, and thus possibly act as promoting agents

---

EXPLANATION OF PLATES.

FIG. 1.—Appearance of experimental animal after depilation and before painting, showing one of the bilaterally symmetrical major zones (A) of active hair growth, and two small minor zones of activity (B and C).

FIG. 2.—Dorsal view of same animal as Fig. 1 after 6 paintings with 9 : 10-dimethyl-1 : 2-benzanthracene, on the left side showing masking of previous zones, prolific hair growth on the painted side and suppression of hair growth on the contralateral side.

FIG. 3.—Photograph of same rabbit as in Fig. 1 after 9 paintings with 9 : 10-dimethyl-1 : 2-benzanthracene showing initial tumour (X) which has occurred in the originally active zone (Fig. 1, A) and a second tumour (Y) which seems to have occurred in an originally small active minor zone (Fig. 1, B).

FIG. 4.—The same animal as in previous photographs, showing preponderance of papillomata in the originally active as compared with quiescent zones (Fig. 1).

FIG. 5.—Section of skin from quiescent zone before painting. Note the scanty hair follicles and thin epidermis. × 45.

FIG. 6.—Section of skin from active zone before painting showing thicker epidermis and groups of hair follicles extending deeply into the dermis. × 45.

FIG. 7.—Section of skin from the painted side after 6 applications of 9 : 10-dimethyl-1 : 2-benzanthracene. Note hyperplastic changes in the epidermis extending into the hair follicles. × 45.

FIG. 8.—High-power view from Fig. 7 showing increased pigmentation in epidermal cells and melanophores in the dermis. × 180.

FIG. 9.—Skin from the non-painted side of an experimental animal (Fig. 7). × 45.

FIG. 10.—Skin from painted side after twelve paintings with 9 : 10-dimethyl-1 : 2-benzanthracene 18 days after appearance of the first papilloma. × 65.

FIG. 11.—Papilloma. × 6.

FIG. 12.—Biopsy from edge of malignant ulcer. × 15.

Whiteley and Ghadially.

7

8

9

10

11

12

Whiteley and Ghadially.

lanoline was selected as a solvent, as it is a relatively inert substance in the sense that it produces no inflammatory reaction, and would not cause masking of the naturally occurring zones of activity.

### SUMMARY.

A band of hair 4 inches wide completely encircling the trunk was removed from 10 rabbits. The depilated skin showed bilaterally symmetrical dark zones which showed prolific hair growth within a few days, the rest of the skin remaining bald. 9 : 10-Dimethyl-1 : 2-benzanthracene in lanoline was painted on the left half of 6 animals; the remaining 4 were painted with lanoline. The following changes were observed:

(1) Within 30 days the painted area in the experimental animals became dark and showed prolific hair growth, while on the opposite side there was suppression of hair growth, the skin becoming bald.

(2) In all animals initial papillomata appeared at the site of an originally active zone. The first papilloma appeared after 53 days.

(3) After the appearance of papillomata hair growth ceased and zones of activity appeared on the opposite side.

(4) The first carcinoma appeared after 81 days at the site of an originally active zone.

It was concluded that (*a*) the naturally occurring zones of increased mitotic activity which continued for some time after the first painting must have acted as a promoting agent; (*b*) it seems more probable that the tumours arose from hair follicles, and not the epidermis, as the former are the site of increased mitotic activity during hair growth.

It gives us great pleasure to express our thanks to Professor H. N. Green for his active interest and constructive criticism during the course of these experiments.

### REFERENCES.

Berenblum, I., and Shubik, P.—(1947*a*) *Brit. J. Cancer*, **1**, 379.—(1947*b*) *Ibid.*, **1**, 383.
Bullough, W. S.—(1950) *Ibid.*, **4**, 329.
*Idem* and Eisa, E. A.—(1950) *Ibid.*, **4**, 321.
Haddow, A., Elson, L. A., Roe, E. M. F., Rudall, K. M., and Timmis, G. M.—(1945) *Nature*, **155**, 379.
Mottram, J. C.—(1944*a*) *J. Path. Bact.*, **56**, 181.—(1944*b*) *Ibid.*, **56**, 391.
Tannenbaum, A.—(1947) 'Approaches to Tumour Chemotherapy.' Washington (Amer. Ass. Adv. Sci.).
Willis, R. A.—(1948) 'Pathology of Tumours,' p. 124. London (Butterworth).